# Pretreated Mesenchymal Stem Cells and Their Secretome: Enhanced Immunotherapeutic Strategies

**DOI:** 10.3390/ijms24021277

**Published:** 2023-01-09

**Authors:** Yuran Su, Chenyu Xu, Wen Cheng, Yanmei Zhao, Lei Sui, Yanhong Zhao

**Affiliations:** 1 Department of Prosthodontics, School and Hospital of Stomatology, Tianjin Medical University, Tianjin 30070, China; 2 Department of Orthodontics, School and Hospital of Stomatology, Tianjin Medical University, Tianjin 30070, China; 3 Institute of Disaster and Emergency Medicine, Tianjin University, Tianjin 30070, China

**Keywords:** mesenchymal stem cells, pretreatment, secretome, immuomodulation, inflammatory diseases

## Abstract

Mesenchymal stem cells (MSCs) with self-renewing, multilineage differentiation and immunomodulatory properties, have been extensively studied in the field of regenerative medicine and proved to have significant therapeutic potential in many different pathological conditions. The role of MSCs mainly depends on their paracrine components, namely secretome. However, the components of MSC-derived secretome are not constant and are affected by the stimulation MSCs are exposed to. Therefore, the content and composition of secretome can be regulated by the pretreatment of MSCs. We summarize the effects of different pretreatments on MSCs and their secretome, focusing on their immunomodulatory properties, in order to provide new insights for the therapeutic application of MSCs and their secretome in inflammatory immune diseases.

## 1. Introduction

Mesenchymal stem cells (MSCs) play important roles in regenerative medicine due to their potential for self-renewal and multi-directional differentiation [1]. MSCs were also found to have strong immunoregulation capabilities caused by the secretion of various signal factors or cell to cell contact [2].

### 1.1. Immunoregulatory Characteristics of MSCs

MSCs exert their immunomodulatory potential by regulating the function of a variety of immune cells, especially monocytes/macrophages [3], T-cells [4], natural killer (NK) cells [5] and B cells [6]. MSCs have been successfully used to treat inflammatory diseases, such as multiple sclerosis [7], diabetes [8], myocardial infarction (MI) [9], graft-versus-host disease (GVHD) [10], Crohn’s disease [11], etc.

According to research, the immunosuppressive properties of MSCs are necessary for restoring immune homeostasis in inflammatory diseases; however, in an active disease state, inflammation frequently changes dynamically, which can alter the immune properties [12]. MSCs can become immunosuppressive under strong inflammation, while weak inflammation enhances their immune responses [13].

The immunomodulatory function of MSCs was initially thought to be intrinsic, but recent studies have shown it is not constitutive and requires some degree of inflammatory response and/or other stimuli, such as an extracellular matrix (ECM) or hypoxia [14,15,16], which is consistent with the stimulations of MSCs in the microenvironment at the inflammatory sites in the human body.

### 1.2. MSC-Derived Secretome

MSCs mainly exhibit their effects due to the expression of various regulatory factors participating in intercellular communication, including cytokines, immune-regulating factors, chemokines, growth factors, nucleic acids, lipids and extracellular vesicles (EVs) in the conditioned media (CM), collectively known as the secretome of MSCs, which is crucial to the regulation of key biological processes [14,17,18]. Using the MSC-derived secretome has the following advantages: (1) It avoids potential safety risks associated with cells transplantation, and so far, no safety issues have been reported in secretome; (2) it offers evaluation of dose, potency and safety which is similar to that of traditional medicine; (3) it allows long-term storage under frozen or freeze-dried conditions without losing biological activity; (4) customized cells lines can be used to produce a large quantity of secretome in controlled laboratory conditions without the need to consider which tissue or donor MSCs should be used; (5) pretreatment of MSCs can significantly increase the content of secretome, with relevant therapeutic effects and disease-specific concerns; (6) the regulation of complex immune response requires the synergistic action of various factors, and the use of a single factor cannot reproduce the immunoregulatory effects of MSCs, so the direct use of secretome avoids component screening [19,20,21,22,23].

Similar to MSCs, the derived secretome is therefore not a constant mixture of secretory factors, but changes depending on the existing microenvironment of MSCs [24]. The function of secretome can be altered differently by the pretreatment approaches, and an appropriate pretreatment may induce MSCs to release a secretome with enhanced regenerative potential [23].

The immunomodulatory capabilities of different pretreatment methods of MSCs and their secretome in innate and adaptive immune responses, as well as in some inflammatory disease models, are the primary focus of this review. In addition, we discuss the problems relating to the application of secretome and the prospect of future research. 

## 2. The Immunomodulatory Effects of Pretreated MSCs and Their Secretome

**Figure 1:**
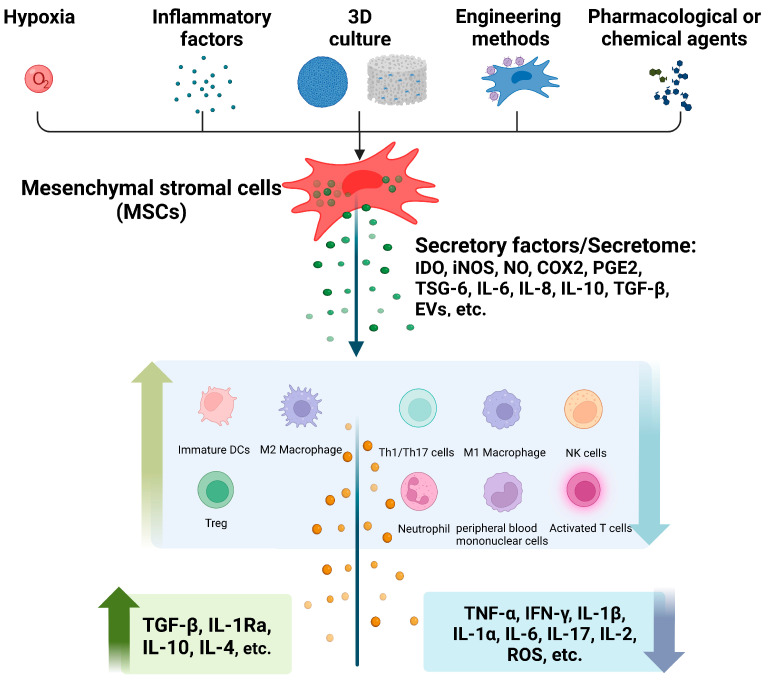
The immunomodulatory effects of different methods pretreated MSCs derived-secretome. Hypoxia, inflammatory factors, 3D culture, engineering methods and pharmacological or chemical agents’ pretreatment induce the release of MSC-derived secretome, then activates or inhibits related multiple immune cells and inflammatory cytokines, ultimately, exert immunomodulatory effects. 3D, three-dimentional; IDO, Indoleamine 2, 3-dioxygenase; iNOS, Inducible nitric oxide synthase; TSG-6, TNF-stimulated gene 6 protein; IL-1α/1β/Ra/2/4/6/8/10/17, Interleukin-1α/1β/1 receptor antagonist/2/4/6/8/10/17; NO, Nitric oxide; COX2, Cyclooxygenase 2; EVs, Extracellular vesicles; DCs, Dendritic cells; Treg, Regulatory T-cell; Th1/17, Type 1/17 helper T; NK, Natural killer; TNF-α, Tumor necrosis factor-α; TGF-β, Transforming growth factor-β; IFN-γ, Interferon-γ; PGE2, Prostaglandin E2; ROS, Reactive oxygen species.

Pretreatment of MSCs prior to application has been performed to increase the immunomodulatory efficacy of MSCs therapy over the past few decades. Many pretreatment strategies, including hypoxia, inflammatory factors, three-dimensional (3D) cell culture, engineering methods and pharmacological or chemical agents, have been used to enhance the immunomodulatory effects of MSCs in many ways (Figure 1).

### 2.1. Hypoxia

MSCs are generally located in a microenvironment of low oxygen (≤ 2–8% O_2_) in vivo, whereas the normoxic oxygen tension used in conventional cells cultures is the atmospheric pressure (21% O_2_) [25], and this variation may have an effect on cells activities. 

Hypoxia is a common feature of the microenvironment of inflammatory tissues, which can profoundly affect the inflammatory process [26]. Hypoxia enhances the immunomodulatory effect of MSCs by increasing the production of cytokines or EVs associated with immunomodulation (Table 1).

Hypoxia-pretreated MSCs could cause peripheral blood mononuclear cells (PBMCs) to undergo apoptosis by boosting the synthesis of the Fas ligand in vitro and reduce local skin inflammation by decreasing tumor necrosis factor-α (TNF-α) while increasing interleukin (IL)-10 [27]. Hypoxia and calcium ions exhibited enhanced immunomodulatory functions of MSCs used to treat GVHD via the monocyte chemoattractant protein-1 (MCP-1) and p53/p21 cascade [28]. The expression of indoleamine 2, 3-dioxygenase (IDO) [29] and inducible nitric oxide synthase (iNOS) [30], mediators of MSCs immunomodulation, increased after hypoxia pretreatment, and then inhibited CD8^+^ T-cell activation and promoted Treg proliferation in vitro. Under 1 and 20% oxygen levels, MSCs were capable of strong upregulation of IDO upon stimulation with interferon-γ (IFN-γ) and TNF-α to inhibit T-cell proliferation in vitro [15]. Interestingly, there was no difference between hypoxia and normoxia in the presence of inflammatory factors. Another in vitro study indicated that a combination of IFN-γ-and hypoxia-pretreated MSCs displayed higher induction of IDO over IFN-γ alone, which was related to the metabolic shift to glycolysis of MSCs caused by hypoxia [31]. The CM containing increased growth factors, IL-6 and IL-8 derived from hypoxia-pretreated MSCs promoted skin wound healing [32]. CM from MSCs cultured in near anoxic (0.1% O_2_) conditions exhibited a significant increased production of vascular endothelial growth factor (VEGF), IL-8, MCP-1 and RANTES while decreased inflammatory cytokines, which indicated its regenerative potential [33]. Intravenous injection of the CM improved neuroinflammation after brain injury by increasing M2 microglia while decreasing M1 phenotypes [34]. Consecutive culture in 1% O_2_ promoted MSCs to secrete immunomodulatory cytokines in vitro, and in acute lung injury (ALI) of mice, their CM could limit lung inflammation by modulating anti-inflammatory polarization of macrophages [35]. Furthermore, a hypoxic environment could promote MSCs to release more EVs, which significantly ameliorated the eosinophils and pro-inflammatory mediators in chronic asthmatic mice [36]. Meanwhile, the EVs could alleviate inflammatory bone loss by transferring miR-210-3p, which directly targets NF-κB1 to induce M2 macrophages polarization and inhibit osteoclastogenesis [37]. Exosomes derived from MSCs under hypoxia promoted functional behavioral recovery of spinal cord injury by shifting microglial polarization from M1 to M2 through miR-216a-5p/TLR4/NF-κB/PI3K/AKT signaling cascades [25]. Proteomics analysis of MSCs and their EVs found the enrichment of biological processes and pathways related to glycolysis, the immune system and extracellular matrix organization in hypoxic condition [38]. 

To sum up, by mimicking low oxygen levels in the inflammatory sites, hypoxic culture pretreatment of the MSC-derived secretome improved its immunomodulatory effects, despite differences in the hypoxic conditions. Beyond that, different tissues and organs in the human body have different levels of oxygen, so different hypoxic pretreatment conditions should be designed according to the specific application in order to better simulate the niche in vivo conditions required for MSCs.

### 2.2. Inflammatory Factors

Aside from hypoxia, the production of inflammatory factors is another simultaneous and related condition in inflammatory diseases, which can actively communicate with the resident MSCs in the tissue microenvironment. Many studies have demonstrated that the pretreatment of MSCs with inflammatory factors could improve their immunomodulatory functions (Table 2).

#### 2.2.1. IFN-γ

IFN-γ is a key pro-inflammatory factor that plays an important role in regulating the immunomodulatory function of MSCs. In 2006, researchers first proposed IFN-γ as a key player in activating the immunosuppressive ability of MSCs, at least in part, by enhancing IDO activity [39]. Then, another study found that IFN-γ enhanced the immunosuppressive activity of MSCs against NK cell activity and protected MSCs from NK-cell-mediated cytotoxicity by promoting the production of IDO and prostaglandin E2 (PGE2) [40]. In addition, subsequent studies found that IDO expression was conducted through the JAK/STAT1 pathway [41] and glucose metabolic reprogramming [89]. Meanwhile, in experimental colitis, mucosal damage was reduced because the pretreated MSCs significantly inhibited type 1 T helper (Th1) inflammatory responses by decreasing T-cell activation and proinflammatory cytokine secretion [42]. Another study indicated that the pretreated MSCs inhibited T-cell proliferation, Th1 cytokine secretion and T-cell degranulation through B7H1 and B7DC/PD1 pathways [43]. Although IFN-γ pretreatment increased IDO activity of MSCs, IDO was unnecessary for inhibition of T-cell function [43]. In addition, the pretreatment significantly increased the factor H secretion of MSCs, and then inhibited the complement activation, suggesting another mechanism of MSCs’ broad immunosuppressive effects [46]. Exosomes derived from pretreated MSCs increased miR-125a and miR-125b, which inhibited the differentiation of Th17 cells and then had therapeutic efficacy for colitis [44]. In addition, pretreated MSC-derived EVs, containing TNF-stimulated gene 6 protein (TSG-6) and A20, inhibited T-cell proliferation [45].

#### 2.2.2. TNF-α

TNF-α, another important pro-inflammatory cytokine, was undoubtedly involved in the immunoregulation of MSCs. CM from TNF-α-pretreated MSCs eased the symptoms of experimental allergic conjunctivitis via cyclooxygenase 2 (COX2)/PGE2 signaling [47]. Furthermore, TNF-α enhanced CD73 expression of MSC-derived exosomes, leading to the polarization of M2 macrophages. Local injection of exosomes significantly reduced the resorption of periodontal bone in mouse periodontitis models and exosomal miR -1260b played an important role in inhibiting the osteoclast activity via the Wnt5a/RANKL pathway [48]. Meanwhile, miRNA-299-3p was upregulated in the pretreated MSC-derived exosomes, which attenuated inflammatory damage of acute liver failure and promoted liver tissue repair [49]. In addition, TNF-α pretreatment could also exert immunosuppressive function by increasing the factor H secretion of MSCs [46].

#### 2.2.3. IL-1β

In inflammatory diseases, IL-1β also acts as a pathological mediator. MSCs pretreated with IL-1β could treat colitis by increasing COX2, IL-6 and IL-8 to regulate the polarization and differentiation of immune cells [50]. Pretreatment of IL-1β resulted in a strong upregulation of miR-146a [51] and miR-21 [52] in MSC-derived exosomes, and this transferred to macrophages, resulting in M2 polarization. Meanwhile, the exosomes significantly inhibited astrogliosis and inflammatory responses via the Nrf-2 signaling pathway [53], and enhanced anti-inflammatory activity via miR-147b mediated inhibition of NF-κB pathway [54]. By upregulating the expression of transforming growth factor-β1 (TGF-β1) and matrix metalloproteinases (MMPs), CM from pretreated MSCs promoted wound healing in vivo [55]. MSCs pretreated with IL-1, including IL-1α and β, increased expression of granulocyte-colony-stimulating factor via IL-1 receptor 1, and the CM decreased the production of inflammatory factors in activated microglial cells [56]. Additionally, by secreting pro-inflammatory cytokines, the secretome from pretreated MSCs regulated the inflammatory response and aggrecan deposition in degenerative intervertebral discs [57].

#### 2.2.4. Lipopolysaccharide (LPS)

LPS is the main cell wall component of Gram-negative bacteria and play a crucial role in the interaction between pathogens and the host immune system [90]. It is also a commonly inflammatory inducer in the study of inflammatory diseases.

It was found that a low concentration of LPS-pretreated MSCs inhibited activated T-cell by up-regulating nitric oxide (NO) [58]. Pretreated MSCs displayed high levels of IL-6, IL-8 and more effectively promoted diabetic skin-flap survival [59]. The CM of pretreated MSCs defended against V. cholerae infection by inducing vibriocidal antibodies and regulating inflammatory and anti-inflammatory effects [60]. Meanwhile, the CM promoted the recruitment and phagocytosis of polymorphonuclear neutrophil granulocytes (PMNs) by upregulating IL-6, IL-8 and macrophage migration inhibitory factor (MIF) of MSCs [64]. Furthermore, LPS pretreatment of MSC-derived exosomes significantly increased anti-inflammatory cytokines while decreased pro-inflammatory cytokines of monocytes and macrophages, promoted their polarization to M2 macrophages and reduced the inflammatory response of diabetic skin wounds and MI [61,62]. The miRlet-7/TLR4/NF-B/STAT3/ATK pathway may be responsible for this effect [61]. Under inflammatory conditions, LPS-pretreated MSC-derived EVs could decrease the RANKL/OPG of PDLSCs by inhibiting the ROS/JNK pathway and promote M2 macrophages polarization through the ROS/ERK pathway, which enhanced the therapeutic efficacy for periodontitis [63].

Some studies have suggested that LPS promotes MSCs to generate a pro-inflammatory phenotype and reverse their therapeutic immunosuppressive effect. Brief LPS pretreatment of MSCs increased IL-6 and IL-8 and showed a decreased capacity to inhibit T cells. It also reversed the protective effect of MSCs against experimental autoimmune encephalomyelitis (EAE) and colitis [65,66]. Meanwhile, pretreated MSC-derived exosomes promoted M1 macrophages polarization, IL-6 and TNF-α production and Th17 cells differentiation [67]. Furthermore, LPS promoted the expression of thrombospondin-1 of MSCs, which was involved in LPS proinflammatory regulation [67].

This immunomodulatory difference of LPS-pretreated MSCs may be caused by cell types (mouse versus human), tissue origin, concentration of LPS, in vivo versus in vitro studies and cells’ pretreatment time. A study indicated that different LPS pretreatment time could change the phenotype of MSCs, playing different immunomodulatory and therapeutic roles [68].

#### 2.2.5. Polyinosinic–Polycytidylic Acid (poly(I:C))

Poly(I:C), a toll-like receptor (TLR) 3 ligand, pretreated MSCs ameliorated colitis by reducing the proliferation of T-cell [66], and improved EAE by inhibiting Th1 and Th17 cells [65]. The pretreated MSCs also promoted ligament healing by promoting M2 macrophages polarization, IL-1Ra expression, early endothelialization and procollagen 1α matrix deposition [91]. In addition, Poly(I:C) pretreatment increased PGE2 production of MSCs via the expression of COX2 and prostaglandin E synthase [69] or Jagged-1-Notch-1 pathway [70], resulting in significant immunosuppression. The above study also found that poly(I:C) induced increased PGE2 secretion compared with IFN-γ or TNF-α [69].

Previous research has observed diverse effects in vitro. TLR4-activated MSCs mostly expressed pro-inflammatory mediators, and TLR3-activated MSCs mostly expressed immunosuppressive factors [92]. This is consistent with the above results of Poly (I:C) pretreated MSCs, while there is still uncertainty related to LPS pretreatment.

#### 2.2.6. Combined Pretreatment of Inflammatory Factors

Combining TNF-α and IFN-γ could also enhance the immunosuppressive activity of MSCs. In normoxic conditions, hypoxia-inducible factor 1α (HIF1α) was induced and maintained by the expression of ROS of activated MSCs [71]. Then, HIF1α enhanced the immunosuppressive activity of MSCs by modulating their metabolic status [71]. The pro-inflammatory effects caused by high levels of palmitate were fully reversed by the pretreated MSCs, which provided a robust and durable prelicensing regimen for protecting MSCs’ immunosuppressive function in relation to type 2 diabetes [72]. Meanwhile, the pretreatment increased IDO production of MSCs and promoted M2 macrophages polarization to inhibit PBMCs [73]. In acute myeloid leukemia therapy, the pretreated MSCs increased tumor necrosis factor-α-related apoptosis-inducing ligand and IDO, enhancing the pro-apoptotic effect of leukemic cells [80]. The CM derived from combined pretreated MSCs efficiently accelerated wound closure via IL-6-dependent M2 polarization [74,75]. Related mechanism research showed that the combined pretreatment increased the production of IDO and TSG-6 by PI3K/AKT pathway, thereby enhanced the immunosuppressive ability of MSCs [76]. Interestingly, another study indicated that MSCs can be induced to generate a pro-inflammatory phenotype by the combined pretreatment, but these MSCs also produced anti-inflammatory factors, which concurred with the classical conception that the two microenvironments inhibiting and promoting tumor growth coexist [77]. The pro-inflammatory chemokines induced aggregation of immune cells closely associated with MSCs, creating a microenvironment in which the local effects produced by MSCs lead to strong immunosuppression. In addition, chaperone-mediated autophagy activation was inhibited in pretreated MSCs, leading to immunosuppressive function [78]. Another study indicated the pretreatment induced miR-155 expression of MSCs and inhibited the immunosuppressive capacity by reducing iNOS expression which might protect MSCs from NO in inflammatory situations [79].

IL-β combined with IFN-γ significantly improved the immunoregulatory effects of MSCs by increasing the production of IDO and PGE2 [81]. Meanwhile, pretreatment of MSCs promoted the secretion of IL-6, inhibited M1 macrophages polarization under inflammation and inhibited M2b polarization under anti-inflammation via the IL-6 pathway [82]. This finding supports the implication that inflammatory condition was a critical determinant of the immunomodulatory characteristics of MSCs described above.

IL-17 could dramatically enhance the immunosuppressive effect of MSCs induced by IFN-γ and TNF-α through the promoted expression of iNOS in liver injury [83] and tumor progression [84].

Compared to the combination of IFN-γ and TNF-α or single treatment, MSCs pretreated by LPS and TNF-α promoted M2 macrophages polarization and enhanced osteogenic differentiation by increasing PGE2 and Arginase-1 [85].

IFN-γ and poly(I:C) pretreated MSCs were more effective at improving experimental colitis. They increased Treg, decreased inflammation and stimulated intestinal epithelial regeneration by promoting the expression of IDO1 [86].

Exosomes derived from MSCs pretreated by TGF-β and IFN-γ promoted the differentiation of Treg, dependent on IDO [87].

MSCs pretreated by IL-1β, IL-6, TNF-α and IFN-γ increased the production of inactive chemerin, which could then be activated by inflammation-induced tissue proteases, thereby promoting the migration of ChemR23- expressing immune cells with immunomodulatory effects [88].

To summarize, many inflammatory factors have been used to pretreat MSCs, and in general, pro-inflammatory pretreatments increased the immunosuppressive properties of MSCs by inducing the anti-inflammatory phenotype of immune cells and stimulating the expression of anti-inflammatory factors. However, due to the fact that there are numerous inflammatory factors and more choices for combined applications, it is difficult to reach unified conclusions or conduct in-depth research in a certain direction. Meanwhile, some studies suggest a combination of different inflammatory factors is superior to a single one, but the other studies directly applied combinations of inflammatory factors and compared them with untreated MSCs. So, it is impossible to verify whether the combination is better than the single factor, which needs more research to confirm this. There is one more point that, so far, there is no clear standard for the selection of combined inflammatory factors.

It is worth noting that MSCs, like immune cells, can retain information from environmental stimuli for a period of time. When pretreated with LPS or TNF-α, MSCs displayed increased secretion of IL-6, IL-8 or MCP-1 and retained high expression of these cytokines for over 7 days without the influence of cell division. These cytokines were increased further after secondary stimulation [59]. Following LPS pretreatment, MSCs increased the production of iNOS, IL-1β and IL-6 after IFN-γ and TNF-α retreatment, and decreased expression of proinflammatory genes after LPS retreatment [93]. Thus, the inflammatory stimuli treated MSC-derived-secretome can contain more abundant regulatory factors. This also suggests that the secondary stimulation of MSC-derived secretome could lead to more effective in immunomodulation, which is worth further exploration.

### 2.3. D Cell Culture

MSCs are typically grown in two-dimensional (2D) culture plates. However, removing them from their endogenous 3D niche, as well as enzymatic passaging, can result in a loss of multipotency, accumulation of chromosomal aberrations within the MSCs genome, dysfunction in DNA repair, replicative senescence, decreased surface markers (e.g., CD105, CD90, CD73) associated with the MSCs’ undifferentiated phenotype [94,95,96,97,98]. Three-dimensional cell culture systems’ ability to bridge the gap between cell-based methods and animal models for studying the repair and replacement of tissues and organs is becoming increasingly apparent [99].

Three-dimensional cell culture methods have been used to improve the immunomodulatory properties of MSCs (Table 3). A study indicated that the loss of T-cell-suppressive abilities in MSC spheroids was caused by increasing PGE2 and decreasing IDO and CD73 [100]. MSC spheroids promoted the paracrine of TSG-6 and ameliorated renal function of acute kidney injury (AKI) compared with 2D-cultured cells [101]. After transplantation into mouse, MSC spheroids promoted the production of IFN-γ and IL-6 while suppressing TNF-α, thereby alleviating liver necrosis and promoting regeneration [102]. MSC spheroids increased the production of immunomodulatory paracrine factors and the immunosuppressive effect on macrophages functional activity, which was further strengthened by pretreatment with IFN-γ and TNF-α [103]. The CM from MSC spheroids efficiently inhibited the expression of inflammatory cytokine, increased the expression of anti-inflammatory cytokines in LPS activated macrophages, and promoted the transformation of M1 into M2 macrophages [104,105,106]. It also had the potential to accelerate wound healing and mature, fully functional tissue regeneration [107]. In addition, the predominant expression of anti-inflammatory cytokines in CM from 3D cultured MSCs demonstrated its superior regenerative and anti-inflammatory potential in arthritis [108] and ear wound model [109]. Compared with 2D culture, spheroid MSC-derived exosomes indicated enhanced immunomodulatory effects by restoring the reactive Th17/Treg balance in inflamed periodontal tissues and experimental colitis [110]. MSC spheroids in combination with hypoxia or hypoxic-mimetic condition provided by dimethyloxalylglycine synergistically increased TSG-6, MMP-2 and VEGF involved in tissue repair processes [111].

MSCs cultured on electrospun gelatine/polycaprolactone fibers scaffolds in advance promoted the expression of IL-6 and chemokines, and the secretome significantly improved corneal wound healing [112]. Synthetic polyisocyanide hydrogel created a 3D culture environment and directed the secretome composition of MSCs, which had therapeutic potential in wound healing [14].

In summation, 3D cultures of MSCs efficiently exerted immunosuppressive effects. However, there was still some debate regarding the MSC spheroids. When they reached critical sizes, their nutrient and oxygen supply might be non-homogenous and could affect cells proliferation and viability [113]. Compared with spherical culture, the secretome produced by 3D hydrogel-cultured MSCs had enhanced immunomodulatory capabilities and regeneration potential [114]. Research has found that the matrix stiffness [115] and composition [14] of hydrogel, and enhanced cell–cell contacts in spheroids [103] could regulate the immunomodulatory function of MSCs. At present, most studies focus on the immune regulation and mechanism of 3D-cultured MSCs, while the interaction between cells has not been thoroughly and comprehensively explored. Studies showed that the microenvironment established within the spheroids acted in an autocrine process to increase the cell-cell interaction [107]. In addition, the matrix stiffness, viscoelasticity, porosity, and degradation of 3D hydrogel and scaffold directly affect MSCs’ aggregation and promote their secretion by increasing cell–cell contacts [116].

Therefore, hydrogel could be designed with suitable structures and properties to ultimately generate an optimized secretome. Meanwhile, as simulated ECM, hydrogels could be used as a tool to collect the secretome of MSCs, which is worthy of further investigation.

### 2.4. Engineered Pretreatment

Engineered MSCs contribute to the immunomodulatory ability by expressing specific immunomodulators (Table 4).

IL-4 transfected MSCs in spheroids demonstrated improved chondroprotective and anti-inflammatory effects in osteoarthritis (OA) model both in vitro and in vivo [117]. IL-10, TGF-β1 or IL-35 transfected MSCs could significantly prolong allograft survival by regulating the production of inflammatory cytokines and phenotypic transformation of immune cells [118,119,120,121]. As an inducible enzyme responsible for the breakdown of haem, haem oxygenase-1 (HO-1) had antioxidant and immunomodulatory functions [136]. Programmed death-ligand 1 (PD-L1) was critical to Treg differentiation and function. HO-1 or IDO overexpressing MSCs and EVs derived from PD-L1 transfected MSCs efficiently improve graft-versus-host reaction following transplantation by increasing Treg and anti-inflammatory cytokines, and decreasing proinflammatory cytokines, NK cells and effector T-cell activity [122,123,124,125,126,127].

Broad-spectrum antibacterial fusion peptide BPI21 and LL-37 transfected MSCs not only enhanced antibacterial and toxin-neutralizing activities but displayed significant immunomodulatory effects [128].

MSCs, overexpressed by HIF1α, exhibited increased resistance to NK-cell-mediated lysis, impaired DCs differentiation and induced higher attraction of monocytes which acquired immunosuppressive properties efficiently [129]. HIF1α-overexpressed MSCs with or without pro-inflammatory treatment released EVs with potent immunomodulatory activity [130,131].

Soluble fibrinogen-like protein 2 (sFgl2), mainly secreted by Treg, exerted potently immunosuppressive activities. sFgl2 overexpressed MSCs inhibited acute heart transplant rejection and induced immune tolerance by promoting M2 macrophage polarization [132].

Hepatocyte growth factor overexpressed MSCs could enhance the treatment effect on psoriasis by regulating T-cell differentiation and inflammatory factors expression [133].

Compared with MSC spheroids pretreated with IFN-γ, heparin microparticle-mediated release of IFN-γ in MSC spheroids could increase sustained immunomodulatory effects by inducing continuous IDO expression throughout 7 days of culture and anti-inflammatory cytokines secretion of monocytes [134]. Similarly, to achieve sustained IDO production of MSCs, the internalization of budesonide -loaded poly (lactic-co-glycolic acid) microparticles into MSCs could improve their immunosuppressive properties [135].

Engineered MSCs have advantages in maximizing and maintaining immunomodulatory potential, and they enable the enhanced expression of specific factors without consideration for which tissues or donors MSCs should derive. Engineered MSCs are mostly directly applied at present, and given that the effect of engineered MSCs is superior to the transfected factor itself, it is speculated that the secretome derived from engineered MSCs is more useful. Subsequent studies should concentrate on the effect of secretome to make the most of its advantages. Meanwhile, the complicated process and the influence of transfection efficiency and stability continue to restrict development. The addition of drug/cytokine-loaded microparticles provides an effective method to enhance and maintain immunomodulatory activity of MSCs. But drug/cytokine selection, sustained release time and dosage are still in need of further study.

### 2.5. Pharmacological or Chemical Agents

Pharmacological or chemical agents, with simple, cheap and efficient features, also represent common pretreatments for MSCs in immunomodulation (Table 5).

Tetrandrine is a potent calcium channel blocker and anti-inflammatory antioxidant agent. It can increase PGE2 secretion of MSCs through the NF-κB/COX2 signaling pathway and significantly reduced pro-inflammatory factors production [137].

Glucocorticoids have seen decades of clinical use as potent anti-inflammatory and immunosuppressive agents. Budesonide increased IDO expression of MSCs following IFN-γ treatment and restored IDO expression in over-passaged MSCs via glucocorticoid-induced expression of transcriptional enhancer Forkhead box O3 (FOXO3) [135]. Pretreatment of MSCs with dexamethasone enhanced immunosuppression in mixed lymphocyte reaction [138]. Dexamethasone and JWH-133, a cannabinoid receptor 2 selective agonist, could restore the anti-inflammatory and immunoregulatory properties of immune thrombocytopenia patient-derived-MSCs via Bcl2 signaling [139].

Melatonin is a ubiquitous hormone involved in the function of immune cells. Melatonin pretreated MSC-derived exosomes promoted M2 macrophages polarization by increasing PTEN expression and inhibiting AKT phosphorylation, and regulated inflammatory factors expression, ultimately promoting the healing of diabetic wounds [140]. Exosomal miRNAs, such as miR-34a, miR-124, and miR-135b may be responsible for the improved anti-inflammatory regulation [141].

With regard to trimetazidine or diazoxide, common anti-ischemic drugs for angina, pretreating MSC-derived secretome increased the secretion of IL-10, TNF-α and IL-1β from LPS-activated PBMCs [142] and reduced systemic proinflammatory cytokines, immune cells recruitment and inflammatory cells infiltration [143].

Pretreated MSCs with chlorzoxazone, a drug currently used for muscle relaxation, were able to significantly suppress T cells, increase IDO and other mediators, and more effectively reduce inflammatory infiltration and tissue damage associated with AKI by modulating the phosphorylation of FOXO3 [144].

Metformin, commonly used to treat type 2 diabetes, optimized the immunoregulatory properties of MSCs by enhancing the production of IDO, IL-10 and TGF-β through the STAT1 pathway, resulting in significant disease activity improvement in lupus [145].

Neuropeptide Substance P (SP) was involved in inflammation regulation and wound healing. By promoting the release of TGF-β1, SP pretreated MSCs might restore the reduced immunosuppressive function of late-passage MSCs, which could improve the therapeutic effectiveness of expanded MSCs in long-term culture [146].

Rapamycin was a macrolide used clinically for anti-fungal treatment and immunoregulation. Rapamycin pretreatment of MSCs mediated T-cell proliferation, migration and differentiation by increasing the secretion of TGF-β1 and CXC-chemokine ligand 8 of MSCs [147,148], and improved the pathological changes, inflammatory cytokine levels and hepatic function in the liver damage model [149]. All of the above effects were achieved by inducing autophagy.

Cytochalasin B (CB) had a reversible effect on the interaction of cytoskeleton and membrane. CB pretreated MSC-derived microvesicles could suppress the production of anti-sheep red blood cell antibody [150], the proliferation of PBMCs and the activation of Th cells, B cells and T-cytotoxic lymphocytes [151].

An IDO metabolite, kynurenic acid, specifically regulated TSG-6 production. Kynurenic acid pretreated MSCs could further promote TSG-6 production and thus enhance the therapeutic capacity of ALI [152].

Glutamine is a nonessential amino acid that modulates immune responses. Glutamine in high doses used to pretreat MSCs modulated immune responses by regulating anti- and pro-inflammatory cytokines, while the CM had immunosuppressive effects on lymphocytes and macrophages [153].

Tongxinluo, a Chinese medicine used to treat coronary artery disease, pretreated MSC-derived exosomes demonstrated better cardioprotective effects by inhibiting apoptosis and inflammation via miR-146a-5p/IRAK1/NF-κB p65 pathway [154].

Magnesium was able to modulate the immunoregulatory properties of MSCs by decreasing pro-inflammatory cytokines and increasing anti-inflammatory cytokines of MSCs stimulated with LPS or TNF-α, and the CM modulated the production profile of inflammatory cytokines in macrophages [155].

To sum up, these studies demonstrated the potentially beneficial effects of MSCs pretreated with pharmacological or chemical agents. This kind of pretreatment can effectively avoid the side effects of drug administration caused by in vivo application, control the drug dosage and improve the efficiency of treatment. The study of drug selection, specific pretreatment conditions and related mechanisms should be carried out in the future.

## 3. Immunomodulatory Mechanisms of Pretreated MSCs and Their Secretome

**Figure 2:**
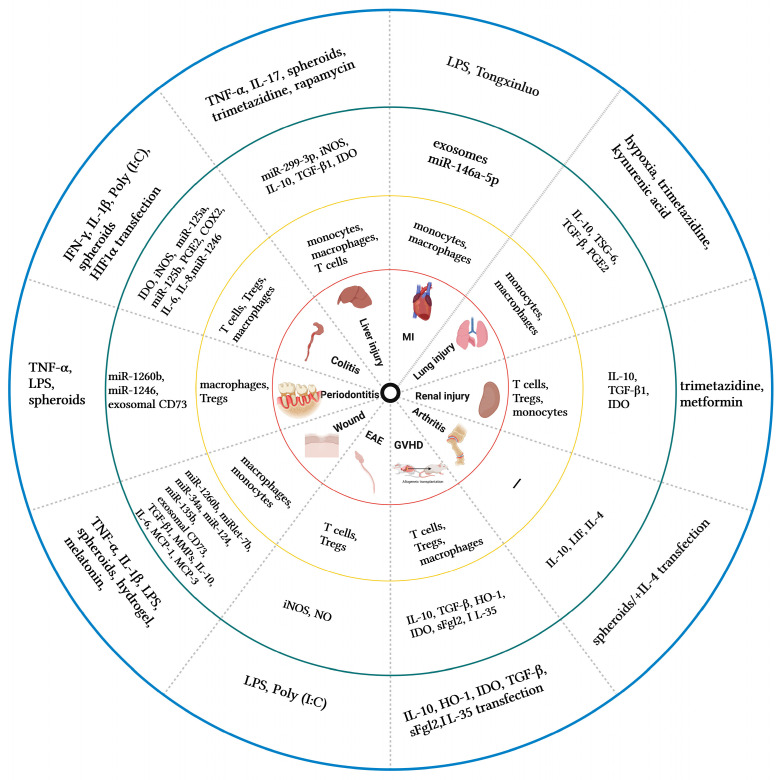
The application of pretreated MSCs and their secretome in multiple inflammatory disease models. The blue circle corresponds to the pretreatment methods, the green circle corresponds to secretory factors, the yellow circle corresponds to related immune cells and the red circle corresponds to the applied inflammatory disease model. IFN-γ, Interferon-γ; TNF-α, Tumor necrosis factor-α;IL-4/6/8/10/1β/17/35, Interleukin-4/6/8/10/1β/17/35; LPS, Lipopolysaccharide; Poly(I,C), Polyinosinic-polycytidylic acid; IDO, Indoleamine 2, 3-dioxygenase; MCP-1/3, Monocyte chemoattractant protein-1/3; iNOS, inducible nitric oxide synthase; miR, Micro ribonucleic acid; NO, Nitric oxide; TGF-β, Transforming growth factor-β; TSG-6, TNF-stimulated gene 6 protein; COX2, Cyclooxygenase 2; HO-1, haem oxygenase-1; PGE2, Prostaglandin E2; GVHD, Graft-versus-host disease; Treg, Regulatory T-cell; LIF, Leukemia inhibitory factor; EAE, experimental autoimmune encephalomyelitis; sFgl2, soluble fibrinogen-like protein 2; MI, Myocardial infarction.

Pretreated MSCs and their secretome modulate inflammatory and immune responses by promoting/inhibiting multiple immune cells and cytokines (Figure 1) and play active roles in different inflammatory disease models (Figure 2). Obviously, several factors play important roles in this process.

### 3.1. IDO/iNOS

IDO, a principal enzyme in tryptophan catabolism, regulated immune responses and promoted cancer progression [156]. IDO catalyzed tryptophan into kynurenine, and following the subsequent enzymatic reactions, kynurenine was further converted into the energetic substrates to fuel cellular metabolic functions [157]. Hypoxia, inflammatory factors, engineering methods and drug pretreatment could increase IDO production of human MSCs to inhibit the proliferation and activation of T-cell [15,31,39,43,66,73,122,130,131,134,144,145], NK cells [39,40], PBMCs [41,42,72,81,135,138] and production of pro-inflammatory cytokines [43,72,86,103,123,130,131,134,135,149], and enhance Treg proliferation [29,81,86,87,122,123,145] and M2 polarization [73,130,131].

The immunosuppressive effects of IDO are related to its characteristic tryptophan catabolism: IDO mediated tryptophan depletion and production of kynurenine, which was involved in the inhibition of immune cells [158]. Tryptophan is an essential amino acid and its reduction would affect the survival of immune cells [159]. Kynurenine could induce T-cell arrest or apoptosis, and its accumulation may induce the differentiation of Treg [160].

Inflammatory factors and engineering pretreatments promoted iNOS expression in the case of rodent MSCs. iNOS catalyzes arginine, resulting in the production of NO, which mediates a variety of biological processes, including immune regulation [161]. NO regulated the immune cells, particularly the proliferation and responsiveness [16,30,42,65,68,83], apoptosis [58], Th1 and Th17 cells differentiation [65,68] in vitro and T-cell infiltration [83], the percentage of Th1 and Th17 subsets [65,68] and inflammatory cytokines production [42] in inflammatory tissue, then consequently regulated the immune response.

The inhibition of T cells may be attributed to the process of NO production depleting L-arginine, which is an essential nutrient for T cells [162]. Meanwhile, NO caused T-cell cycle arrest by affecting the JAK signal transductor and STAT signal pathway. In addition, NO could also regulate MAPK and NF-κB [163]. Furthermore, NO induced Treg which inhibited T cells and Th17 cells in vitro and in vivo [164], interfered with the production of pro-inflammatory cytokines in macrophages [165], and attenuated the cytotoxicity and capacity of NK cells to produce inflammatory cytokines [166]. Additionally, iNOS attenuated inflammation by reducing the influx of DCs and T cells and their ability to produce inflammatory cytokines [167].

Researchers also identified a common downstream mechanism of T-cell inhibition in both IDO and iNOS as endoplasmic-reticulum stress [168].

### 3.2. COX2/PGE2

PGE2, a mediator of many physiological and pathological functions, and COX2, a key enzyme that catalyzed the rate-limiting steps in the conversion of AAs to prostaglandins, both play crucial roles in the inflammatory process [169]. Consecutive hypoxia, inflammatory factors, spheroid culture and pharmacological pretreatment effectively promote COX2/PGE2 expression. The COX2/PGE2 axis effectively promoted M2 macrophages polarization [35,50,85,105,106,170]. A study indicated that COX2-dependent production of PGE2 was a major paracrine mediator of MSCs in regulating M1 to M2 macrophages polarization by changing their metabolic status [171]. In addition, PGE2 reprogramed macrophages by acting on their EP2 and EP4 receptors of them and stimulated the expression of IL-10 [172].

Meanwhile, COX2/PGE2 expression of pretreated MSCs also inhibited the proliferation, migration and activation of T cells [81,100,144,173], proliferation of mononuclear cells [70,174] and PBMCs [81,138], activity of NK cells [40], differentiation of Th1 and Th17 cells [50,70,81] and the secretion of pro-inflammatory cytokines [70,103,137,155,173,174,175], and enhanced Treg differentiation [70,81,170,174,175]. In addition, PGE2 could modulate the differentiation of DCs toward an anti-inflammatory and reparative profile [176], and suppressed the differentiation of monocytes to mature DCs in vitro [177].

### 3.3. TSG-6

TSG-6 was an inflammation-associated protein secreted by MSCs, which mediated immunomodulation and regeneration [178]. Spheroid culture and some drug pretreatment promoted TSG-6 expression of MSCs. TSG-6 inhibited the migration of neutrophils via direct interaction with the neutrophils chemokine CXC-chemokine ligand 8 [179], and then inhibited the neutrophils infiltration in injury tissue [152]. Meanwhile, TSG-6 induced M1 to M2 macrophages polarization [35,106] and inhibited T-cell [45,144] and TNF-α secretion of activated macrophages [104]. Furthermore, TSG-6 also increased the production of Treg and suppressed the Th1 mediated immune response [180]. Collectively, TSG-6 derived from pretreated MSCs effectively inhibited inflammation response in different inflammatory models [101,104,144,152]. Importantly, IDO could mediate the regulation of TSG-6 expression through its metabolite, kynurenic acid [152].

### 3.4. MSC-Derived EVs

MSC-derived EVs regulated the biological function of immune cells by transferring multiple types of bioactive cargos, such as nucleic acids and proteins to targeting cells. Local and systemic application efficiently inhibited detrimental immune responses and promoted regeneration in inflamed tissues [181].

The immunomodulatory effects of pretreated MSC-derived EVs depended on the transfer of miRNAs (e.g., miR-216a-5p, miR-125a, miR125b, miR-1260b, miR-146a, miR-146a-5p, miR-210-3p, miR-34a, miR-124, miR-299-3p, miR-21, miR-147b, miRlet-7b, miR1246 and miR-135b) and proteins (e.g., TGF-β, CD73, IFN-γ, PD-L1, IDO, A20, TSG-6 and IL-10) to target cells and then enabled their phenotypic conversion into immunosuppressive types [25,37,44,48,49,51,52,54,61,62,63,87,110,140,141]. EVs also acted on T cells and PBMCs [45,87,127,130,131,150,151] and inhibited ROS in MSCs [63].

### 3.5. IL-6

IL-6 is a pleiotropic cytokine involved in inflammation, immune response and hematopoiesis [182]. Pretreated MSCs exhibited different expression of IL-6 and different immunomodulatory properties, because IL-6 had context-dependent pro- and anti-inflammatory properties [183]. It has been suggested that the regenerative or anti-inflammatory effects of IL-6 were mediated by classic signaling whereas its pro-inflammatory effects were mediated by trans-signaling [184].

IL-6 increased following several different pretreatment methods. On one hand, high expression of IL-6 might play a positive role in the recruitment and migration of monocytes, macrophages and MSCs, and then promote wound healing [32,59,64,102,107,112]; on the other hand, it might have anti-inflammatory effects [50,57,66,71,75,103,129,130,131,170,185]. However, due to the multiple simultaneously occurring factors after pretreatment, the actual function of IL-6 was not clarified.

Here, we try to focus on its anti-inflammatory mechanisms. IL-6, to a lesser extent, could modulate the in vitro differentiation of macrophages and DCs toward an anti-inflammatory and reparative profile [176]. IL-6 could enhance macrophages differentiation into M2 subtype by activating JAK2/STAT3 signaling pathway [186], upregulate IL-4 receptor expression, promote STAT6 phosphorylation of macrophages, and promote M2 macrophages polarization [75,187]. Additionally, IL-6 promoted the production of IL-10 of T cells, which would restrict many inflammatory processes [188].

As a variable component of the secretome, IL-6 may be a keystone cytokine in regulating inflammatory states and deserves more attention.

## 4. Problems and Prospects

Earlier studies primarily attributed the therapeutic effect of MSCs to their ability to locally transplant and differentiate into multiple tissues, but the implanted cells are unable to survive for a long period of time [189]. Additionally, the direct application of MSCs is constrained in a few ways, including by the limited source of cells, immunocompatibility, tumorigenicity, emboli formation, phenotype changes in differentiation and proliferation, and problems related to cells’ treatment, storage and transportation, as well as the time-consuming process of cell culture, senescence, sensitivity to toxic environments, and heterogeneity in isolation and culture methods between different laboratories [21,25,190,191,192,193,194,195].

By contrast, using the MSC-derived secretome as a cell-free therapy strategy can help to effectively avoid the abovementioned problems and provides several advantages over MSC-based applications which have described in “Introduction”.

Studies have shown that pretreating MSC-derived secretome reveals immunomodulatory potential more efficiently than in untreated MSCs. Compared to engineered methods, pretreating MSCs with drugs, cytokines, hypoxia or 3D culture may be faster and simpler approaches. Additionally, these types of induction techniques more closely the in situ stimulations of inflammatory pathological locations in the host. However, the accompanying issues should be tackled before putting the secretome to clinical use:

1. Selection of pretreatment methods: it is essential to understand how each pretreatment method affects MSCs’ paracrine behavior. Some studies suggest that the combination of different pretreatment methods is superior to one single method, but further studies are still required to confirm this. Meanwhile, pretreatment strategies can be categorized as selective or non-selective: selective approaches, such as engineered methods, target a single pathway or a small number of related pathways to achieve a desired secretome, whereas non-selective strategies, such as hypoxia or inflammatory factors, activate multiple signaling pathways that collectively increase expression of downstream signal factors or receptors.
2. Heterogeneity: it is extremely difficult to understand the mechanism underlying the reported effects on pretreatment of MSCs due to their tissue origin, the health and age of the donors, cells separation and culture techniques and the animal models.
3. Purification: the current method for collecting and purifying secretome is centrifugation to remove the cell debris in CM [112], and some studies concentrated the secretome after centrifugation [108,143]. However, additional culture media components may be collected simultaneously and may influence the purity of secretome. Currently, there is no standard purification method of secretome as there is for exosomes.
4. Components selection: the comparative effect of the specific components (soluble factors or EVs) and the overall application of secretome remains to be verified.
5. Standardization and optimization: this is problematic because secretome is a combination of various molecules and deserves further study to evaluate its potency and determine a safe dosage.

Collectively, in the subsequent studies, optimal design of pretreating and reliable isolation methods needs to be developed to purify the secretome; cell culture and cell line selection need to be unified. A simple and reliable method of standardizing secretome features is also required.

## 5. Conclusions

MSCs exhibit a regulatory phenotype and react quickly to the environmental stimulus that influence their secretome. Pretreatment of MSCs with hypoxia, inflammatory factors, 3D culture, engineering methods and pharmaceutical stimuli, or a combination of the above methods prior to application is a novel strategy to enhance the immunomodulatory effects of MSCs and their secretome in the local or systemic immune response. These models showed that it was possible to improve the regulation of innate and adaptive immune responses. The variable approaches and combinations employed in MSC pretreatment need to be optimized, and new techniques need to be developed to better characterize and standardize the secretome. In the near future, we believe this strategy may be studied further and potentially applied to treat individuals with immune system-related disorders.

## Figures and Tables

**Table 1:** Hypoxia pretreatment of MSCs.

Treatment Conditions	MSCs Sources	Secreted Factors or Expressed Genes	Research Scenarios	Immunomodulatory Effects (Signaling Pathway)	Reference
2% O_2_ 24 h	GT-MSCs	FasL, IL-10↑ TNF-α↓	PBMCs proliferation in vitro skin wound model of mice	PBMCs apoptosis↑ inflammatory cells↓, skin wound closure ↑	[27]
3% O_2_ + calcium (1.8 mmol/L)	UC- MSCs	PLK1↑	T-cell proliferation in vitro GVHD model of mice	T-cell proliferation↓ survival↑, weight loss↓ and histopathologic injuries↓ in GVHD target organs in vivo (MCP-1 and p53/p21)	[28]
5% O_2_	BM-MSCs	IDO↑	T-cell differentiation in vitro	Treg proliferation↑	[29]
1% O_2_ 48 h	BM-MSCs	iNOS, NO↑	inflammatory bowel disease model of mice	CD8^+^ T-cell activation↓ body weight loss, colon shortening and colon inflammation↓	[30]
1% O_2_ + IFN-γ (50 ng/mL) + TNF-α (20 ng/mL) 6, 24, 72 h	AT-MSCs	IDO, CXCL10↑	T-cell proliferation in vitro	inhibition of CD4+ and CD8^+^ T-cell↑ in vitro	[15]
1% O_2_ + IFN-γ (100 ng/mL) 48 h	AT-MSCs	IDO, HLA-G↑	mixed lymphocyte reactions in vitro	T-cell inhibition↑	[31]
2% O_2_	BM-MSCs	FGF, VEGF, IL-6 and IL-8↑	monocytes migration in vitro skin wound model of mice	CD14^+^ monocyte migration↑ macrophages recruitment↑	[32]
0.1% O_2_ 7 d	BM-MSCs	IL-8, VEGF, MCP-1, RANTES↑ IL-6, IL-1β, IL-15, IL-1Ra↓	CM implanting subcutaneously in mice	inflammatory effect↓ after subcutaneous transplantation in vivo	[33]
5% O_2_ 24 h	AT-MSCs	CM	traumatic brain injury model of rats	neurological impairment and cognitive deficiency↓, neuroinflammatory edema and nerve fiber damage↓, M1 macrophages↓ and M2 macrophages↑, IL-6, TNF-α↓ and TSG-6, TGF-β↑	[34]
1% O_2_ 48 h	UC-MSCs	IGF1, IL-10, TSG-6, TGF-β and PGE2↑ IL-6, IL-8↓	lung injury model of mice	anti-inflammatory polarization and efferocytosis of macrophages↑	[35]
5% O_2_ 24 h	UC-MSCs	EVs (miR-146a-5p) ↑	allergic asthma model of mice	eosinophils↓and IL-4, IL-13↓	[36]
1% O_2_ 48 h	DP-MSCs	EVs (miR-210-3p) ↑	calvarial osteolysis model of mice osteoclast differentiation and macrophages polarization in vitro	inflammatory bone loss↓ in vivo M2 polarization↑ and osteoclastogenesis↓ in vivo and in vitro (NF-κB1)	[37]
1% O_2_ 48 h	BM-MSCs	exosomal miR-216a-5p↑	contusive spinal cord injury model of mouse microglia/macrophages polarization in vitro	functional behavioral recovery after spinal cord injury↑ in vivo M1 to M2 microglia/macrophages polarization↑ in vivo and in vitro (TLR4/NF-κB/PI3K/AKT)	[25]

Abbreviations: O_2_, Oxygen; IFN-γ, Interferon-γ; h, Hours; d, Days; TNF-α, Tumor necrosis factor-α; MSCs, Mesenchymal stem cells; BM/GT/UCAT/DP-MSCs, Bone marrow/Gingival tissue/Umbilical cord/Adipose tissue/Dental pulp-derived-MSCs; IL-6/8/10/1β/1Ra/15/4/13, Interleukin-6/8/10/1β/1receptor antagonist/15/4/13; FasL, Fas ligand; PLK1, Polo-like kinase 1; IDO, Indoleamine 2, 3-dioxygenase; HLA-G, Human leukocyte antigen-G; iNOS, inducible nitric oxide synthase; CXCL10, CXC-chemokine ligand10; NO, Nitric oxide; MCP-1, Monocyte chemoattractant protein-1; TGF-β, Transforming growth factor-β; CM, conditioned medium; EVs, Extracellular vesicles; IGF1, Insulin-like growth factor 1; miR, Micro ribonucleic acid; TSG-6, TNF-stimulated gene 6 protein; PGE2, Prostaglandin E2; PBMCs, Peripheral blood mononuclear cells; GVHD, Graft-versus-host disease; Treg, Regulatory T-cell; TLR4, Toll-like receptor 4; NF-κB, Nuclear factor-κB; PI3K, phosphoinositide 3-kinase; AKT, Protein kinase B; ↑, increased expression; ↓, decreased expression.

**Table 2:** Inflammatory factors pretreatment of MSCs.

Treatment Conditions	MSCs Sources	Secreted Factors or Expressed Genes	Research Scenarios	Immunomodulatory Effects (Signaling Pathway)	Reference
IFN-γ (2 ng/mL)	BM-MSCs	IDO↑	T, NK cells proliferation in vitro	proliferation of activated T or NK cells↓	[39]
IFN-γ (50 ng/m) 48 h	BM-MSCs	IDO, PGE2↑	NK cells activation in vitro	NK cells activation↓	[40]
IFN-γ (200 IU/mL) 24 h	BM-MSCs UC-MSCs AT-MSCs WJ-MSCs	IDO↑	PBMCs proliferation in vitro GVHD model of mice	PBMCs proliferation↓ immune cells infiltration in skin and small intestine↓ and survival rate↑ of GVHD mice (JAK/STAT1)	[41]
IFN-γ (500 U/mL)	BM-MSCs	IDO, iNOS↑	PBMCs proliferation in vitro colitis model of mice	PBMCs proliferation↓ serum amyloid A protein levels and local proinflammatory cytokine levels↓ in colonic tissues	[42]
IFN-γ (50 ng/mL) 72 h	BM-MSCs	IDO (independent)↑, B7H1, B7DC↑	T-cell proliferation and cytokines production in vitro	T-cell proliferation↓ Th1 cytokine (IFN-γ, TNF-α and IL-2) ↓ T-cell degranulation↓ (B7H1 and B7DC/PD1)	[43]
IFN-γ (50 ng/mL)	BM-MSCs	Exosomal miR-125a and miR-125b↑	T-cell differentiation in vitro colitis model of mice	differentiation of Treg↑ and Th17 cells ↓in vitro body weight loss, disease activity index, colon shortening, impaired intestinal structure, TNF-α, IFN-γ, IL-6 and Th 17 cells↓ and Treg↑ in vivo (Stat3)	[44]
IFN-γ (10 ng/mL)/ TNF-α (15 ng/mL) 72 h	AT-MSCs	EVs (A20 and TSG-6) ↑ RAB27B	T-cell proliferation in vitro	activated CD4^+^ T-cell↓	[45]
IFN-γ (10 and 100 ng/mL) 12, 24, 48 h/ TNF-α (100 ng/mL) 12, 24, 48 h	MSCs	H factor↑	modified sheep erythrocytes hemolytic assay in vitro	complement activation↓	[46]
TNF-α (10 ng/mL) 48 h	BM-MSCs	COX2, PGE2↑	experimental allergic conjunctivitis model of mice	TNF-a, IL-4, IL-5, IL-1β↓and TGF-β↑in the conjunctiva B-cell IgE release↓ activation of mast cells↓ histamine release↓ (COX2/PGE2)	[47]
TNF-α (100 ng/mL) 48 h	GT-MSCs	exosomal miR-1260b, exosomal CD73↑	macrophages polarization in vitro skin wound model of mice periodontitis model of mice	M2 macrophages polarization↑ in vitro and in vivo wound healing↑ TRAP^+^ osteoclasts and bone resorption↓ (Wnt5a/RANKL)	[48]
TNF-α (1 ng/mL) 72 h	UC-MSCs	exsomal miR-299-3p↑	LPS-activated Kupffer cells cytokines production in vitro acute liver failure model of mice	secretion of IL-1β, IL-18 and IL-6 in Kupffer cells↓ the damage of liver tissue↓, IL-6, IL-1β, IL-18, aspartate aminotransferase and alanine aminotransferase in peripheral blood serum↓ of acute liver failure mice (NLRP3)	[49]
IL-1β (10 ng/mL) 48 h	UC-MSCs	COX2, IL-6 and IL-8↑	colitis model of mice	M1 macrophages↓ in the peritoneal cavity of mice Th1, Th17↓ and Treg, Th2↑ in spleen and lymph nodes	[50]
IL-1β (10 ng/mL) 12 h	UC-MSCs	exosomal miR-146a↑	macrophages polarization in vitro sepsis model of mice	M2 macrophages polarization↑ in vitro and in vivo survival rate↑, TNF-α, IL-6↓ and IL-10↑ in serum of septic mice	[51].
IL-1β (10 ng/mL) 12 h	MSCs	exosomal miR-21↑	macrophages polarization in vitro sepsis model of mice	M2 macrophages polarization↑ in vitro and in vivo survival rate↑, TNF-α↓ and IL-10↑ in serum of septic mice	[52]
IL-1β (10 ng/mL) 24 h	BM-MSCs	exosome	LPS-activated astrogliosis in vitro status epilepticus model of mice	astrogliosis↓ C3, CD81 and Ki67↓, BDNF, IL-1Ra, VEGF, IL-10 and NGF↑ in vitro and in vivo (Nrf-2)	[53]
IL-1β (25 ng/mL) 24 h	BM-MSCs	exosomal miR-147b	IL-1β-induced inflammatory SW982 cells cytokines production in vitro	IL-1β, IL-6, and MCP-1↓ in inflammatory SW982 cells (NF-κB)	[54]
IL-1β (1 ng/mL) 24 h	GT-MSCs	TGF-β1, MMP-1, MMP-9↑	LPS-activated THP-1 cytokines production in vitro skin wound model of mice	TNF-α↓ and IL-1Ra↑ in THP-1 skin wound healing↑	[55]
IL-1β (10 ng/mL) 24 h	BM-MSCs	G-CSF↑	LPS-treated BV2 microglial cells cytokines production in vitro	IL-6, TNF-α↓ and IL-10↑ of BV2 microglial cells	[56]
IL-1β (10 ng/mL) + 6 % O_2_ 48 h	BM-MSCs	IL-6, RANTES, IL-8, MCP-1 and PGE2↑	degenerative intervertebral disc model of bovina in vitro	IL-6, IL-8↓, aggrecan↑ in intervertebral disc	[57]
LPS (0.1 μg/mL) 12 h	BM-MSCs	NO↑	activated T-cell apoptosis in vitro	apoptosis of T-cell↑	[58]
LPS (100 ng/Ml) 24 h	AT-MSCs	IL-6 and IL-8↑	skin flap model of diabetic rats	skin flap survival↑ in diabetic rats	[59]
LPS (5 µg/mL) 72 h	MSCs	CM	immunized model of mice	serum IL-4, IL-5, BAFF, TGF-β↑ and TNF-α↓ in humoral immunity CD4^+^ T-cell↑ serum IgG, saliva IgA↑ in immunized mice serum IL-6, TNF-α, IL-10↑ in immunized mice vibriocidal activity↑	[60]
LPS (100 ng/mL) 48 h	UC-MSCs	exosomal miRlet-7b↑	macrophages polarization in vitro cutaneous wound model of diabetic rats	M2 macrophages polarization↑ in vitro and in vivo M1 macrophages and inflammation↓ in diabetic wounds (TLR4/NF-κB/STAT3/AKT)	[61]
LPS (100 ng/mL) 24 h	BM-MSCs	exosomes	macrophages polarization and cytokines production in vitro myocardial infarction model of mice	M2↑ and M1↓ in vitro and in vivo IL-6, TNF-α, IL 1β↓ and IL-10↑of macrophages post-infarction inflammation↓ (NF-κB/AKT1/AKT2)	[62]
LPS (250 ng/mL) 24 h	DF-MSCs	EVs	periodontitis model of beagle dogs	ROS and RANKL/OPG ratio↓ of LPS pretreated PDLSCs (ROS/JNK) M2 macrophages polarization↑ (ROS/ERK) periodontal tissue regeneration↑ of periodontitis dog	[63]
LPS (10 ng/mL) 24 h	BM-MSCs PG-MSCs	IL-6, IL-8, MIF↑	anti-microbial PMNs activity in vitro	migration of PMNs↑ CCL4 secretion of PMNs↑ (NF-κB) anti-microbial activity of PMNs↑	[64]
LPS (500 ng/mL) 1 h	BM-MSCs	NO↓ IL-6, IL-8↑	T-cell proliferation and differentiation in vitro EAE model of mice	proliferation of CD3^+^ T-cell, Th1 and Th17↑ percentages of Th1 and Th17↑ in EAE mice reverse the therapeutic immunosuppressive effect of MSCs	[65]
LPS (10 ng/mL) 1 h	UC-MSCs	IL-6, IL-8↑	T-cell proliferation in vitro colitis model of mice	T-cell proliferation↑ clinical signs and severity of intestinal inflammation↑ in vivo	[66]
LPS (1 μg/mL), 72 h	T-MSCs	thrombospondin-1↑ exosomes	macrophages polarization, cytokines production and T-cell differentiation in vitro	M1 macrophages, IL-6, TNF-α↑ differentiation of CD4^+^ T-cell to Th17 cells↑	[67]
LPS (500 ng/mL) 48 h	BM-MSCs	IL6, iNOS↑	T-cell proliferation in vitro EAE model of mice	T-cell proliferation↓ clinical score↓, Th17↓ and Treg↑ in vivo	[68]
Poly (I:C)) (10 μg/mL) 1 h	BM-MSCs	NO↑ IL-6↓	T-cell proliferation and differentiation in vitro EAE model of mice	proliferation of CD3^+^ T-cell, Th1 and Th17↓ percentages of Th1 and Th17↓ in EAE mice clinical signs and the severity↓ of EAE	[65]
Poly (I:C)) (1 μg/mL) 1 h	UC-MSCs	IDO, IL-6 and IL-8↑	T-cell proliferation in vitro colitis model of mice	T-cell proliferation↓ clinical signs and severity of intestinal inflammation↓ in the acute murine model of colitis	[66]
Poly (I:C)) (100 μg/mL) 24 h	BM-MSCs	COX2, PGES↑	GVHD model of mice	(TLR3/PGES/COX2/PGE2)	[69]
Poly (I:C)) (1 μg/mL)	UC-MSCs	PGE2↑	colitis model of mice	IFN-γ, IL-17A, IL-21, IL-23↓ and IL-10↑ in colitis tissues proliferation of activated mesenteric lymphnode cells↓ Th1/17 cells↓ and Treg↑ in the colon proliferation of mononuclear cells↓ clinical and pathological manifestations in colitic mice↓ (TLR3/Jagged-1/Notch-1)	[70]
TNF-α + IFN-γ 24 h	BM-MSCs	ROS, HIF1α↑	delayed-type hypersensitivity model of mice	oxidative phosphorylation metabolism↓ Th1, Th17↓in vitro and in vivo	[71]
TNF-α (1 ng/mL) + IFN-γ (10 ng/mL) 24 h	MSCs	IDO↑ PTGS2 and IL-6↓ in the presence of palmitate	PBMCs proliferation and cytokines production in vitro	PBMCs proliferation and production of IFN-γ, TNF-α↓ in the presence of palmitate (IFN-γ/JAK1/2)	[72]
TNF-α (3 ng/mL) + IFN-γ (10 ng/mL) 24 h	BM-MSCs	IDO↑	macrophages polerization and T-cell proliferation in vitro	M2 macrophages↑ T-cell proliferation↓	[73]
TNF-α (20 ng/mL) + IFN-γ (20 ng/mL) 24 h	UC-MSCs	CM	macrophages migration, polarization and cytokines production in vitro skin wound model of mice	migration, M2 polarization, and phagocytic ability of macrophages↑ secretion of VEGF, IL-10, IL-13 and IL-4↑ and TNF-α↓ of macrophages proangiogenic ability↑ wound closure↑ (IL-6/IL-4Rα/STAT6/PPARγ and IL-6/STAT3)	[74,75]
TNF-α (10 ng/mL) + IFN-γ (10 ng/mL) 24 h	UC-MSCs	IDO, TSG-6↑ CXCL9, CXCL10 and CXCL11↑ Hexokinase II↑	inflammatory bowel disease model of mice	inflammatory parameters in inflammatory bowel diseases mice↓ (PI3K/AKT)	[76]
TNF-α (1.5 ng/15 ng) + IFN-γ (6.5 ng/65 ng) 48 h	BM-MSCs	CCL5, CXCL9, CXCL10 and CXCL11↑ IDO, PD-L1 and HLA-G↑	/	/	[77]
TNF-α (10 ng/mL) + IFN-γ (10 ng/mL) 24 h	BM-MSCs	CMA, LAMP-2A↓	T-cell proliferation and recruitment t in vitro inflammatory liver injury model of mice	T-cell↓ in vitro and in vivo T-cell recruitment to MSCs↑ (NF-κB and STAT1/AKT)	[78]
TNF-α (10 ng/mL) + IFN-γ (10 ng/mL) 24 h	BM-MSCs	miR-155↑	T-cell proliferation in vitro	T-cell proliferation↓ iNOS and NO expression of MSCs↓ (TAB2)	[79]
TNF-α (20 ng/mL) + IFN-γ (50 ng/mL) 24 h	UC-MSCs	tumor necrosis factor-α-related apoptosis-inducing ligand and IDO↑	leukemia cells cycle, apoptosis in vitro acute myeloid leukemia model of mice	leukemia cells apoptosis↑ survival↑, leukemia burden in peripheral blood and bone marrow↓ of acute myeloid leukemia mice	[80]
IL-1β (5 ng/mL) + IFN-γ (20 ng/mL) 24 h	UC-MSCs	PGE2, IDO↑	PBMC proliferation, T-cell differentiation and migration in vitro colitis model of mice	PBMCs proliferation↓ Th1 differentiation↓ and Treg differentiation↑ CD4 ^+^ T-cell migration↑ body weight, colon structure improvement↑	[81]
IL-1β (3 ng/mL) + IFN-γ (30ng/mL) 24 h	BM-MSCs	NO, IL-6 and PGE2↑	macrophages polarization in vitro	M1 macrophages↓ in inflammation M2b macrophages↓ in anti- inflammation (IL-6)	[82]
IL-17 (10 ng/mL) + IFN-γ (10 ng/mL) + TNF-α (10 ng/mL) 12 h	BM-MSCs	iNOS↑	T-cell proliferation in vitro hepatitis model of mice	T-cell proliferation↓ mononuclear cells and T-cell infiltration↓ in liver injury mouse model	[83]
IL-17 (10 ng/mL) + IFN-γ (10 ng/mL) + TNF-α (10 ng/mL) 24 h	BM-MSCs	iNOS, NO↑	tumor model of mice	tumor growth↑ (NO/STAT3)	[84]
LPS (20 μg/mL) + TNF-α (20 ng/mL) 72 h	BM-MSCs	PGE2 and Arginase-1↑	macrophages polarization in vitro	M2 macrophages↑ M1 macrophages↓	[85]
IFN-γ (100 ng/mL) + poly(I:C) (10 μg/mL)	BM-MSCs	IDO1↑	colitis model of mice	body weight loss, colon shortening and colon epithelial loss, crypt destruction, inflammatory cells infiltration↓, intestinal stem cells proliferation, enterocyte differentiation, and epithelial regeneration↑ in inflammatory bowel diseases mice IL-1β, TNF-α,, IL-6↓, IL-10↑ in colon tissue Treg↑ in lymphoid organs and in vitro	[86]
TGF-β (10 ng/mL) + IFN-γ (1000 IU/mL) 72 h	UC-MSCs	exosomes (IL-10, IFN-γ, IDO) ↑	PBMCs proliferation and differentiation in vitro	PBMCs proliferation↓ proportion of Treg↑	[87]
IL-1β (50 ng/mL) + IL-6 (40 ng/mL) + TNF-α (100 ng/mL) + IFN-γ (200 ng/mL) 72 h	BM-MSCs	chemerin↑	L1.2-ChemR23 cells migration in vitro	L1.2-ChemR23 cells migration↑	[88]

Abbreviations: IFN-γ, Interferon-γ; h, Hour/ Hours; TNF-α, Tumor necrosis factor-α; LPS, Lipopolysaccharide; IL-1β/17/6/8/10//2/4/5/13/18/1Ra/4Rα/21/23, Interleukin-1β/17/6/8/10//2/4/5/13/18/ 1receptor antagonist /4 receptor α/21/23; poly(I,C), Polyinosinic-polycytidylic acid; COX2, Cyclooxygenase 2; TGF-β, Transforming growth factor-β; PGE2, Prostaglandin E2; MSCs, Mesenchymal stem cells; BM/UC/AT/WJ/GT/DF/PG-MSCs, Bone marrow/Umbilical cord/Adipose tissue/Wharton’s jelly/Gingival tissue/Dental follicle/Parotid gland-derived-MSCs; T-MSCs, Thymic MSCs; IDO, Indoleamine 2, 3-dioxygenase; CCL4/5, CC-chemokine ligand4/5; iNOS, Inducible nitric oxide synthase; miR, Micro ribonucleic acid; MMP-1/9, Matrix metalloproteinases-1/9; G-CSF, Granulocyte-colony stimulating factor; MIF, Macrophage migration inhibitory factor; RANTES, regulated on activation, normal T cell expressed and secreted; NO, Nitric oxide; EVs, Extracellular vesicles; CM, Conditioned media; TSG-6, TNF-stimulated gene 6 protein; PGES, Prostaglandin synthases; ROS, Reactive oxygen species; PTGS2, Prostaglandin-endoperoxidase synthase 2; HIF1α, Hypoxia inducible factor 1α; CXCL9/10/11, CXC-chemokine ligand 9/10/11; PD-L1, Programmed death ligand-1; CMA, Chaperone mediated autophagy; LAMP-2A, CMA related lysosomal receptor lysosomal-associated membrane protein 2; HLA-G, Human leukocyte antigen-G; NF-κB, Nuclear factor-κB; NK, Natural killer; PBMCs, Peripheral blood mononuclear cells; MCP-1, Monocyte chemoattractant protein-1; VEGF, Vascular endothelial growth factor; GVHD, Graft-versus-host disease; JAK, Janus kinase; STAT1/3/6, Signal transducers and activators of transcription 1/3/6; Th1/2/17, Type 1/2/17 T helper; IgE/G/A, Immunoglobulin E/G/A/; TRAP^+^, tartrate-resistant acid phosphatase positive; RANKL, Receptor activator of NF-κB ligand; BAFF, B-cell activating factor; TLR3/4, Toll-like receptor 3/4; AKT, Protein kinase B; OPG, Osteoprotegerin; JNK, Jun N-terminal kinase; PDLSCs, Periodontal ligament stem cells; Treg, Regulatory T-cell; ERK, Extracellular signal-regulated kinase; PMNs, Polymorphonuclear neutrophil granulocytes; EAE, Experimental autoimmune encephalomyelitis; TAB2, TAK1-binding protein 2; PI3K, phosphoinositide 3-kinase; NLRP3, NOD-like receptor family pyrin domain containing 3; NGF, Nerve growth factor; BNDF, brain-derived neurotrophic factor; ↑, increased expression; ↓, decreased expression.

**Table 3:** 3D culture pretreatment of MSCs.

Treatment Conditions	MSCs Sources	Secreted Factors or Expressed Genes	Research Scenarios	Immunomodulatory Effects (Signaling Pathway)	Reference
spheroids	BM-MSCs UC-MSCs	COX2, PGE2↑ CD73, Kynurenine and free phosphate↓	PBMC proliferation in vitro	T-cell suppressive abilities↓	[100]
	AT-MSCs	TSG-6↑	acute kidney injury model of rats	therapeutic effects of AKI↑	[101]
	BM-MSCs	TSG-6, stanniocalcin-1↑	LPS-activated macrophages cytokines production in vitro Peritonitis model of mice	secretion of TNF-α↓of macrophages neutrophil activity, TNF-α, IL-1β, CXCL2/MIP-2, PGE2↓in inflammatory exudates and plasmin activity↓ in serum	[104]
	BM-MSCs	PGE2↑	LPS-activated macrophages macrophages polarization and cytokines production in vitro	TNF-α, IL-6, IL-12P40, IL-23, CXCL2↓ and IL-1Ra, IL-10↑ in macrophages polarization of M1 to M2↑ (COX2/PGE2/EP4) (caspases/NF-κB)	[105]
	BM-MSCs	IL-1α, IL-1β, COX2, TSG-6, stanniocalcin-1 and PGE2↑	macrophages polarization in vitro	polarization of M1 to M2↑ (caspases/NF-κB/IL-1/PGE2 and Notch/PGE2)	[106]
	UC-MSCs	MMP-2, MMP-9, TGF-β1, IL-6, G-CSF↑	skin wound model of rats	wound healing↑ in skin wound	[107]
	UC-MSCs	IFN-γ, IL-6 ↑ TNF-α↓	acute liver failure model of mice	necrosis↓, regeneration↑ and liver repair↑	[102]
	BM-MSCs	PGE2, TGF-β, IDO and IL-6↑	lipopolysaccharide and IFN-γ activated macrophages in vitro	TNF-a secretion↓ of macrophages	[103]
	DP-MSCs	exosomes exosomal miR-1246	periodontitis and colitis model of mice	restored Th17 cells/Treg balance in both the inflamed periodontium and colon alveolar bone loss↓, inflammatory cells↓ and osteoclasts↓ in experimental periodontitis colon length↑, inflammatory cells↓, IL-1β, IL-6, TNF-α↓ in colitis (miR-1246/Nfat5)	[110]
	UC-MSCs	IL-10, LIF↑	arthritis model of rats	local and systemic arthritic manifestations↓	[108]
spheroids +microbeads+ hydrogel	AT-MSCs	IL-10, TGF-β↑	ear full thickness wound model of rabbits	infiltration of lymphocytes↓ in wound ear full-thickness wound healing↑	[109]
spheroids + 2% O_2_/ dimethyloxalylglycine	BM-MSCs	TSG-6, MMP-2 and VEGF↑	tube formation assay in vitro	tube formation of HUVECs↑	[111]
polycaprolactone and gelatin electrospun fiber scaffolds	BM-MSCs	eotaxin, IL-6, LIF, MCP-1 and MCP-3↑	corneal wound model of rabbits in vitro	corneal wound healing↑	[112]
polyisocyanide hydrogel	AT-MSCs	IL-10↑	wound healing assay in vitro	wound healing↑	[14]

Abbreviations: O_2,_ Oxygen; MSCs, Mesenchymal stem cells; BM/UC/AT/DP/-MSCs, Bone marrow/Umbilical cord/Adipose tissue/Dental pulp-derived-MSCs; COX2, Cyclooxygenase 2; PGE2, Prostaglandin E2; VEGF, Vascular endothelial growth factor; TSG-6, TNF-stimulated gene 6 protein; IL-1α/1β/6/10/12p40/23/Ra, Interleukin-1α/1β/6/10/12p40/23/1receptor antagonist; MMP-2/9, Matrix metalloproteinases-2/9; LIF, Leukemia inhibitory factor; TGF-β, Transforming growth factor-β; G-CSF, Granulocyte-colony stimulating factor; IFN-γ, Interferon-γ; TNF-α, Tumor necrosis factor-α; IDO, Indoleamine 2, 3-dioxygenase; miR, Micro ribonucleic acid; MCP-1/3, Monocyte chemoattractant protein-1/3; AKI, Acute kidney injury; LPS, Lipopolysaccharide; CXCL2, CXC-chemokine ligand 2; Th17:Type 17 T helper; MIP-2, Macrophage inflammatory protein-2; Treg: Regulatory T-cell; EP4, Prostaglandin E receptor subtype 4; Nfat5: Nuclear factor of activated T-cell 5; HUVECs, Human umbilical vein endothelial cells; ↑, increased expression; ↓, decreased expression.

**Table 4:** Engineering pretreatment of MSCs.

Treatment Conditions	MSCs Sources	Secreted Factors or Expressed Genes	Research Scenarios	Immunomodulatory Effects (Signaling Pathway)	Reference
IL-4 gene transfection +spheroids	AT-MSCs	IL-4↑	IL-1β-treated chondrocytes apoptosis and cytokines production in vitro osteoarthritis model of rats	chondrocytes apoptosis↓, NO, iNOS MMP-13)↓and cartilage ECM (Col2) ↑ in chondrocytes production of NO↓, chondrocyte apoptosis↓, expression of the pain mediators↓ in osteoarthritis	[117]
IL-10 gene transfection	MSCs	IL-10↑	orthotopic liver transplantation model of rats	liver allograft survival↑ acute rejection↓ Th17↓ and Treg↑ IL-17, IL-23, IL-6, IFN-γ, TNF-α↓ and IL-10, TGF-β1↑ in T-cell of liver and in serum	[118]
	BM-MSCs	IL-10↑	T-cell proliferation in vitro corneal allograft model of rats	proliferation of T-cell↓ corneal allograft survival time↑ infiltration of CD4^+^, CD68^+^ T-cell↓ in the corneal grafts CD4^+^, CD68^+^ T-cell↓ and Treg↑ in the draining lymph nodes lncRNA 003946 expression↑ in CD68^+^ infiltrating cells	[119]
TGF-β1 gene transfection	BM-MSCs	TGF-β1↑	T-cell proliferation, cytokines production and differentiation in vitro liver allograft model of rats	T-cell proliferation and IFN-γ secretion↓ and Treg↑ acute rejection↓ and survival↑ after liver transplant T-cell, Th17 cells, IL-1β, IL-6, IFN-γ↓, Treg, IL-10↑ in vivo	[120]
IL-35 gene transfection	AT-MSCs	IL-35↑	cardiac allograft model of mice	acute cellular rejection↓ allograft survival↑ Th17, Th1/Th2↓ and Treg↑ in spleen IL-17↓ in graft	[121]
IDO gene transfection	BM-MSCs	IDO↑	T-cell proliferation, differentiation and cytokines production in vitro orthotopic renal transplantation model of rabbits	T-cell proliferation↓, Treg↑, CTLA-4, IL-10 and TGF-β1 expression of Treg↑ in vitro renal graft survival and tolerance↑	[122]
	BM-MSCs	IDO↑	T-cell and DCs differentiation in vitro heterotopic heart transplantation model of rats	DCs markers↓ and Treg↑ in vitro and in vivo IL-10, TGF-β↑ and IL-2, IFN-γ↓ in serum infiltration of inflammatory cells, hemorrhage, edema, and myocardial damage↓ in the transplantation mode	[123]
HO-1	BM-MSC	HO-1↑	small bowel allograft model of rats	recipient survival rates↑ clinical manifestation and weight loss↓ grading of acute rejection↓ in small bowel graft apoptotic↓ cells in small intestine mucosa↓ NK cells activity↓ in graft IFN-γ. IL-2, IL-17, IL-6, IL-23, TNF-α↓ and IL-10, TGF-β↑ in serum Treg↑ in the spleen	[124]
	BM-MSC	HO-1↑	orthotopic liver transplantation model of rats	recipient survival rates and liver function↑ clinical manifestations↓ grading of acute rejection↓ in hepatic grafts apoptotic cells↓ in hepatic tissue IFN-γ. IL-2, IL-17, IL-6, IL-23, TNF-α↓ and IL-10, TGF-β↑ in serum Treg↑ in the spleen	[125]
	BM-MSC	HO-1↑	lymphocytes proliferation and differentiation in vitro reduced-size liver transplantation model of rats	T-cell, NK cells activation↓ and Treg↑ in vitro recipient survival rates↑ clinical manifestations↓ grading of acute rejection↓ in hepatic graft Treg↑ in the spleen TNF-α, IL-2, IL-17, IL-23↓ and IL-10, TGF-β↑ in serum NK cells activation↓ in hepatic graft	[126]
PD-L1 gene transfection	BM-MSC	EVs (PD-L1)	T-cell proliferation, differentiation and cytokines production in vitro GVHD model	Treg↑, T-cell proliferation↓ IL-10, IL-2, TGF-β, IFN-γ↑ of CD4^+^ T-cell host versus graft rejection↓ graft survival↑ Treg↑ and Th17, Th1 cells↓ in vivo	[127]
BPI21/LL-37 gene transfection	UC-MSCs	BPI21/LL-37↑	antibacterial activity and endotoxin neutralization assay LPS-activated macrophages in vitro sepsis model of mice	antibacterial and endotoxin-neutralizing activity↑ IL-1β, TNF-α, IL-6↓ and IL-10↑ both in macrophages and in serum bacterial clearance and endotoxin-neutralizing↑ in septic mice	[128]
HIF1α gene transfection	DP-MSCs	HIF1α, CCL2/MCP-1, galectin 1, IL-6↑	T-cell proliferation, DCs differentiation, monocytes recruitment and differentiation, and NK cells-mediated lysis in vitro	T-cell proferization↓ DCs differentiation↓ recruitment of monocytes and differentiation into suppressor macrophages↑ degranulation and IFN-γ production of NK cells↓	[129]
HIF1α gene transfection/+ IFN-γ (50 ng/mL) + TNF-α (10 ng/mL) + IL-1β (10 ng/mL)	DP-MSCs	EVs IL-6, IDO↑	macrophages polarization, T-cell proliferation, PBMCs adhesion in vitro delayed type hypersensitivity model of mice colitis model of mice	M1 repolarizes to M2↑ efferocytic and immunosuppressive capacity of M1↑ fibrosis Induced by TGF-β expression of VCAM and P-selectin of HUVECs↓ PBMCs adhesion on activated endothelium↓ proliferation of CD4^+^, CD8^+^ T-cell↓ leucocyte infiltration↓ ear-swelling response↓ hyperplasia↓ and CD45^+^ cells infiltration↓, M1↓and M2↑ in a DTH mice inflammatory cells infiltration↓, TNF-α, IL-1β, IL-6↓, M1↓and M2↑ in colitis mouse model (PD-L1/PD-1/NF-κB)	[130,131]
sFgl2 gene transfection	AT-MSCs	sFgl2↑	IFN-γ and LPS-activated macrophages polarization, migration and phagocytosis in vitro heart transplantation model of mice	M1↓, M2↑ in vitro and in vivo (JAK/STAT and NF-κB) phagocytosis and migration of macrophages↑ myocyte necrosis, vasculitis, lymphocytes infiltration↓ in the heart grafts Treg↑ in spleen IFN-γ, IL-12, TNF-α, IL-6, and IL-1β↓, TGF-β1, IL-4, IL-10↑ in the serum acute rejection after heart transplantation↓	[132]
hepatocyte growth factor gene transfection	DP-MSCs	hepatocyte growth factor↑	T-cell differentiation in vitro psoriasis model of mice	Th1, Th17 cells↓, Treg↑ expression of cytokeratin 6 and cytokeratin 17↓ in the psoriatic skin lesions. IFN-γ, IL-17A, TNF-α↓ in the serums T-box transcription factor 21, IFN-γ, retinoic acid-related orphan receptor-γt, IL-17A, IL-17F, IL-23↓ and Foxp3, IL-10↑ in the psoriatic skin lesions.	[133]
heparin microparticle loaded with IFN-γ (20 µg/mg microparticle) + spheroids	BM-MSCs	IDO↑	T-cell proliferation, activation and cytokines production in vitro	T-cell activation and proliferation↓ secretion of TNF-α↓, IL-10↑ of PBMCs	[134]
internalization of PLGA microparticle loaded with budesonide (loading 7.05%)	MSCs	IDO↑	PBMCs proliferation and cytokines production in vitro	proliferation and IFN-γ production of PBMCs↓ (STAT1/ FOXO3)	[135]

Abbreviations: IFN-γ; Interferon-γ; IL-2/4/10/35/6/17/23/1β, Interleukin-2/4/10/35/6/17/23/1β; TGF-β, Transforming growth factor-β; IDO, Indoleamine 2, 3-dioxygenase; HO-1, haem oxygenase-1; HIF1α, Hypoxia inducible factor 1α; sFgl2, soluble fibrinogen-like protein 2; PLGA, poly (lactic-co-glycolic acid); MSCs, Mesenchymal stem cells; AT/BM/UC/DP-MSCs, Adipose tissue/Bone marrow/Umbilical cord/Dental pulp-derived-MSCs; CCL2, CC-chemokine ligand 2; MCP-1, Monocyte chemoattractant protein-1; ECM, Extracellular matrix; Col2, Collagen 2; NO, Nitric oxide; lncRNA, Long non-coding RNA; iNOS, Inducible nitric oxide synthase; MMP-13, Matrix metalloproteinases-13; MI, Myocardial infarction; Th1/2/17, Type 1/2/17 T helper; CTLA-4, Cytotoxic T lymphocyte antigen-4; Treg, Regulatory T-cell; DCs, Dendritic cells; NK, Natural killer; STAT1, Signal transducers and activators of transcription1; PD-L1, Programed death ligand-1; VCAM, Vascular cell adhesion molecule; HUVECs, Human umbilical vein endothelial cells; PD-1, Programmed cell death protein 1; DTH, Delayed type hypersensitivity; PBMCs, Peripheral blood mononuclear cells; NF-κB, Nuclear factor-κB; JAK, Janus kinase; LPS, Lipopolysaccharide; FOXO3, Forkhead box O3; GVHD, Graft-versus-host disease; ↑, increased expression; ↓, decreased expression.

**Table 5:** Pharmacological or chemical agent pretreatment of MSCs.

Treatment Conditions	MSCs Sources	Secreted Factors or Expressed Genes	Research Scenarios	Immunomodulatory Effects (Signaling Pathway)	Reference
tetrandrine (5 μM and 10 μM) 24 h	BM-MSCs	PGE2↑	LPS-activated macrophages cytokine production in vitro ear skin inflammation model of mice	TNF-α secretion of LPS-activated macrophages↓ TNF-α↓ in ear skin inflammation sites (NF-κB/COX2)	[137]
budesonide (1 μM) 24 h + IFN-γ (100ng/mL) 48 h	MSCs	IDO↑	PBMCs proliferation and cytokines production in vitro	proliferation and IFN-γ production of PBMCs↓ (STAT1/ FOXO3)	[135]
dexamethasone (1000 ng/mL, 2000 ng/mL, 3000 ng/mL) 24 h and 48 h	UC-MSCs DP-MSCs AT-MSCs BM-MSCs	PGE2, IDO, HLA-G↑	PBMCs proliferationt in vitro	proliferation of PBMCs↓	[138]
JWH-133 (2.5µM) / + dexamethasone (100 nM) 24 h	ITP-MSCs	IL-6↓ IL-4, Bcl2↑	T lymphocytes proliferation and cytokines production in vitro	T lymphocytes proliferation↓ TNF-α↓ in LPS-treated T-cell (Bcl2)	[139]
melatonin (1 μM) 48 h	BM-MSCs	exosomes exosomal miR-34a, miR-124 and miR-135b↑	monocytes polarization and cytokines production in vitro air pouch model of mice diabetic wound healing of rats	ratio of M2 to M1↑ in vitro and in vivo IL-1β, TNF-α, iNOS↓, Arginase-1, IL-10↑ of macrophages in vitro gene expression of TGF-β1, Il-10 and TSG-6↑ of activated THP-1 cells angiogenesis and collagen synthesis↑ in diabetic wound (PTEN/AKT)	[140]
melatonin (10 μM) 72 h	BM-MSCs	exosomal miR-34a, miR-124 and miR-135b↑	monocytes polarization and cytokines production in vitro	M2 polarization and gene expression of TGF-β1, Il-10 and TSG-6↑of activated THP-1 cells	[141]
trimetazidine (50 μM) 6 h /diazoxide (100 μM) 0.5 h	ESC-MSCs	secretome	LPS-activated PBMCS cytokines production in vitro endotoxemia model of mice	IL-10, TNF-α and IL-1β↑ secreted by PBMCs CXCL13, IL-12, CCL2, TNFR1, IL↓ and IL-3, IL-10, KC, CXCL2α, XCL1, CCL5↑ in serum of LPS injected mice necroinflammatory score↓ in kidney and liver of LPS injected mice alveolar space↑ and inflammatory infiltration↓ in lung of LPS injected mice	[142,143]
chlorzoxazone (10 μM) 24 h	UC-MSCs	IDO, COX2, IL-4, TSG-6, CCL5, CXCL9 and CXCL10↑ IL-6↓	T-cell proliferation acute nephritis model of mice	T-cell activation and proliferation↓ inflammatory infiltration and tissue damage in AKI rat model↓ (FOXO3)	[144]
metformin (0.1, 1 and 5 mM) 72 h	AT-MSCs	IDO, IL-10 and TGF-β↑	T-cell proliferation in vitro lupus model of mice	CD4^+^ T-cell proliferation↓ cervical lymph node and kidney weight, proteinuria, serum anti-dsDNA IgG and renal pathology↓ in lupus nephritis mice regulatory effect on peripheral blood and splenic cellular subsets in lupus nephritis mice Th17/Treg ratio↓ of spleen and kidney in lupus nephritis mice	[145]
SP (100 nM) 48 h	BM-MSCs	TGF-β1↑	T-cell proliferation and cytokines production in vitro	activity and IL-2/ IFN-γ secretion of T-cell↓	[146]
rapamycin (3 μM) 24 h	UC-MSCs	TGF-β1↑	T-cell proliferation in vitro	CD4^+^ T-cell proliferation↓	[147]
	BM-MSCs	TGF-β1, CXCL8↑	T-cell migration and differentiation in vitro	migration and Treg differentiation↑ Th1 cells differentiation and IL-17A, IFN-γ, IL-2 production of CD3/CD28^+^ T-cell↓	[148]
	UC-MSCs	IL-10, TGF-β1, IDO↑	liver ischemia/reperfusion injury model of mice	neutrophils infiltration and ROS↓ in liver tissues IL-1β, IL-6, TNF-α gene↓ in liver tissues	[149]
CB (10 µg/mL) 0.5 h	AT-MSCs	microvesicles	PBMCs proliferation and cytokine production, T-cytotoxic lymphocytes, Th cells, and B cells proliferation in vitro transplantation sheep red blood cells immunization model of mice allogeneic and xnogeneic microvesicles in mice	anti-sheep red blood cells antibody↓ in serum proliferation of PBMCs↓, activation of Th cells. B cells and T-cytotoxic lymphocytes↓ fractalkine↓, G-CSF, GM-CSF, MCP-3, MDC, IL-12p70, IL-1β, MCP-1 of PBMCs↑	[150,151]
kynurenic acid (200 μM) 48 h	UC-MSCs	TSG-6↑	acute lung injury model of mice	neutrophil infiltration in ALI↓ (AhR)	[152]
glutamine (2 mM and 10 mM) 14 d	BM-MSCs	IL-1β, IL-6↓ IL-10, TGF-β↑	lymphocytes and macrophages proliferation and cytokine production in vitro	lymphocytes and macrophages proliferation↓ IL-10 production↑ of lymphocytes and macrophages IFN-γ production↓ of lymphocytes (NF-κB/STAT3)	[153]
Tongxinluo (400 μg/mL) 24 h	BM-MSCs	exosomal miR-146a-5p	acute myocardial infarction model of rats	cardiomyocyte apoptosis↓ apoptotic cardiomyocytes, Bax, cleaved-Caspase 3, IL-6, TNF-α, infarct size and cardiac fibrosis↓ and angiogenesis↑ in the infarct region (IRAK1/NF-κB p65)	[154]
Magnesium (5 mM) 24 h + LPS (1.25 μg/mL) /TNF-α (10 ng/mL) 2 h	C3H/10T1/2 MSCs	IL-1β, IL-6↓ IL-10, PGE2↑	LPS-activated macrophages proliferation and cytokine production, lymphocytes proliferation and cytokine production in vitro	Proliferation production of TNF-α, IL-β, IL-6 ↓ and IL-10↑ of macrophages IL-10 production↑ of lymphocytes (NF-κB/STAT3)	[155]

Abbreviations: μM, μmol/L; mM, mmol/L; nM, nmol/L; h, Hours; d, Days; IFN-γ, Interferon-γ; SP, Substance P; TNF-α, Tumor necrosis factor-α; CB, cytochalasin B; LPS, Lipopolysaccharide; MSCs, Mesenchymal stem cells; BM/UC/DP/AT/ITP/ESC-MSCs, Bone marrow/Umbilical cord/Dental pulp/Adipose tissue/Immune thrombocytopenia patients/embryonic-derived-MSCs; A-MSCs, Amniotic MSCs; PGE2, Prostaglandin E2; IL-6/4/10/1β/12/3/2/12p70/1α, Interleukin-6/4/10/1β/12/3/2/12p70/1α; IDO, Indoleamine 2, 3-dioxygenase; Bcl2, B-cell lymphoma 2; HLA-G, Human leukocyte antigen-G; miR, Micro ribonucleic acid; COX2, Cyclooxygenase 2; TSG-6, TNF-stimulated gene 6 protein; CCL2/5, CC-chemokine ligand 2/5; CXCL2α/8/9/10/13, CXC-chemokine ligand 2α/8/9/10/13; HIF1α, Hypoxia inducible factor 1α; MMP-2/9, Matrix metalloproteinases-2/9; TGF-β1, Transforming growth factor-β1; NF-κB, Nuclear factor-κB; TNF-α, Tumor necrosis factor-α; PBMCs, Peripheral blood mononuclear cells; FOXO3, Forkhead box O3; STAT1/3, Signal transducers and activators of transcription 1/3; iNOS, Inducible nitric oxide synthase; MCP-1/3, Monocyte chemoattractant protein-1/3; PTEN, Phosphatase and tensin homolog; AKT, Protein kinase B; KC, keratinocyte-derived-chemokine; XCL1, chemokine C-motif-ligand-1; TNFR1, tumor necrosis factor receptor 1; AKI, Acute kidney injury; ALI, Acute lung injury; ROS, Reactive oxygen species; Th, T helper; G-CSF, Granulocyte-colony stimulating factor; Treg, Regulatory T-cell; GM-CSF, Granulocyte-macrophage colony-stimulating factor; MDC, macrophage-derived-chemokine; ↑, increased expression; ↓, decreased expression.

## Data Availability

Not applicable.
